# Hybrid PET/MRI enables high-spatial resolution, quantitative imaging of amyloid plaques in an Alzheimer’s disease mouse model

**DOI:** 10.1038/s41598-020-67284-z

**Published:** 2020-06-25

**Authors:** Georgia R. Frost, Valerie Longo, Thomas Li, Lauren A. Jonas, Martin Judenhofer, Simon Cherry, Jason Koutcher, Carl Lekaye, Pat Zanzonico, Yue-Ming Li

**Affiliations:** 1 Chemical Biology Program, Memorial Sloan Kettering Cancer Center, New York, NY 10065 USA; 2 Program of Neuroscience, Weill Graduate School of Medical Sciences of Cornell University, New York, NY 10021 USA; 3 Program of Pharmacology, Weill Graduate School of Medical Sciences of Cornell University, New York, NY 10021 USA; 4 Small Animal Imaging Core Facility, Memorial Sloan Kettering Cancer Center, New York, NY 10065 USA; 5 Department of Biomedical Engineering, University of California, Davis, CA 95616 USA; 6 Departments of Medical Physics, Memorial Sloan Kettering Cancer Center, New York, NY 10065 USA; 7 Departments of Radiology, Memorial Sloan Kettering Cancer Center, New York, NY 10065 USA

**Keywords:** Neuroscience, Diseases of the nervous system, Alzheimer's disease

## Abstract

The emergence of PET probes for amyloid plaques and neurofibrillary tangles, hallmarks of Alzheimer disease (AD), enables monitoring of pathology in AD mouse models. However, small-animal PET imaging is limited by coarse spatial resolution. We have installed a custom-fabricated PET insert into our small-animal MRI instrument and used PET/MRI hybrid imaging to define regions of amyloid vulnerability in 5xFAD mice. We compared fluorine-18 [^18^F]-Florbetapir uptake in the 5xFAD brain by dedicated small-animal PET/MRI and PET/CT to validate the quantitative measurement of PET/MRI. Next, we used PET/MRI to define uptake in six brain regions. As expected, uptake was comparable to wild-type in the cerebellum and elevated in the cortex and hippocampus, regions implicated in AD. Interestingly, uptake was highest in the thalamus, a region often overlooked in AD studies. Development of small-animal PET/MRI enables tracking of brain region-specific pathology in mouse models, which may prove invaluable to understanding AD progression and therapeutic development.

## Introduction

Pathologically, Alzheimer’s disease (AD), the most common form of dementia, is marked by the presence of extracellular amyloid plaques in the brain^1^. These plaques are composed of β-amyloid (Aβ), a peptide released from amyloid precursor protein (APP) after sequential cleavage by β-secretase and then γ-secretase^2^. According to the amyloid cascade hypothesis, Aβ accumulation is a causative factor in AD development^3^. Aβ aggregates are thought to trigger a cascade of reactions involving neurofibrillary tangle (NFT) formation, neuronal death, neuroinflammation and, ultimately, cognitive decline. This hypothesis has significant genetic support. For example, familial AD (FAD) mutations have been identified in APP^4^ and presenilin (PS)^5,6^, the catalytic subunit of GS^7–9^. As a defining feature of AD, plaques have been targeted for development of diagnostic agents.

[^18^F]-FDDNP (2-(1-{6-[(2-[F]Fluoroethylidene)malononitrile) was the first radiotracer used for amyloid imaging in AD patients^10^. AD subjects had 10–20% higher uptake in the brain compared to controls^11^. Subsequently, carbon-11-labeled Pittsburgh compound B, [^11^C]-PIB, a derivative of the amyloid dye, Thioflavin T, was shown to have higher specific binding to amyloid compared to [^18^F]-FDDNP^12^. There are, however, conflicting results regarding the use of [^11^C]-PIB to quantify amyloid load in AD mouse models^13,14^. Additionally, [^11^C]-PIB use is restricted to centers with a cyclotron due to the short physical half-life (only 20 minutes) of the ^11^C label. To overcome this, at least three radiofluorinated radiotracers, [^18^F]-Florbetapir^15^, [^18^F]-Florbetapen^16^, and [^18^F]-Flutemetamol^17^, have been developed; ^18^F has a physical half-life of 110 minutes, long enough for wide distribution from regional cyclotron facilities. [^18^F]-Florbetapir has been approved by the US Food and Drug Administration (FDA) and the European Medicines Agency for use in dementia patients.

AD animal models that express a FAD mutation (APP and/or PS1) have been widely used for investigation of Aβ and AD pathogenesis. However, PET imaging of plaques in mouse models is limited by relatively coarse spatial resolution (typically 1 to 2 mm full-width half-maximum (FWHM)) and therefore cannot reliably define specific regions within the mouse brain. To overcome this PET/CT images may be manually co-registered with a sequential MRI scan^18^ or co-registered to an MRI template^19^. Alternatively, PET/CT scans may be used to approximate larger regions such as the cortex or cerebellum^20^.

[^18^F]-Florbetapir has been used to analyze amyloid load and its spatial distribution in APP/PS1-21 mice both *ex vivo* by autoradiography and *in vivo* by microPET. [^18^F]-FC119S (2-[2-(N-monomethyl)aminopyridine-6-yl]-6-[(S)-3-fluoro-2-hydroxypropoxy]benzothazole) also specifically binds to Aβ in the APP/PS1 mouse model^21^, pronounced localization was detected in the cortex, hippocampus and striatum^22^. Furthermore, while too low to be demonstrable visually, localization of [^18^F]-Florbetapir was 14.5% greater in 5XFAD mice, which coexpress five FAD mutations that drive Aβ production^23^, compared to WT mice^24^.

Hybrid PET and MRI technology allows truly simultaneous PET and MRI and has emerged as an appealing means for correlation of tracer uptake and anatomy^25^. Its clinical availability offers immediate benefit for translational research. Combined PET/MR infuses the exquisite PET radiotracer sensitivity with a wide variety of targets and MRI’s superior soft tissue contrast and versatile functional imaging abilities. True simultaneous PET/MRI opens the door to simultaneous dynamic studies in both PET and MRI allowing for acquisition of complementary dynamic parameters from both PET and MRI neurological studies. For example, in the older hybrid technology of PET/computed tomography (CT) (PET-CT), the PET and CT imaging are performed sequentially and thus are temporally as well as spatially offset. In contrast, the true simultaneity of PET and MRI achievable with these new hybrid systems eliminates the misregistration effect of subtle motions between the two scans. Such misregistration can confound correlation of tracer uptake and anatomy, especially on the spatial scale of the mouse brain and regions therein. Of course, MRI offers exquisite contrast among soft tissues, such as different regions of the brain – detail which is simply not achievable with CT. PET-MRI thus offers high-spatial resolution, high-contrast anatomical imaging that complements absolute quantitative tracer uptake obtained with PET. Therefore, PET-MRI would be invaluable to studies utilizing AD mouse models as it enables quantification of tracer uptake within different regions of the brain.

**Figure 1:**
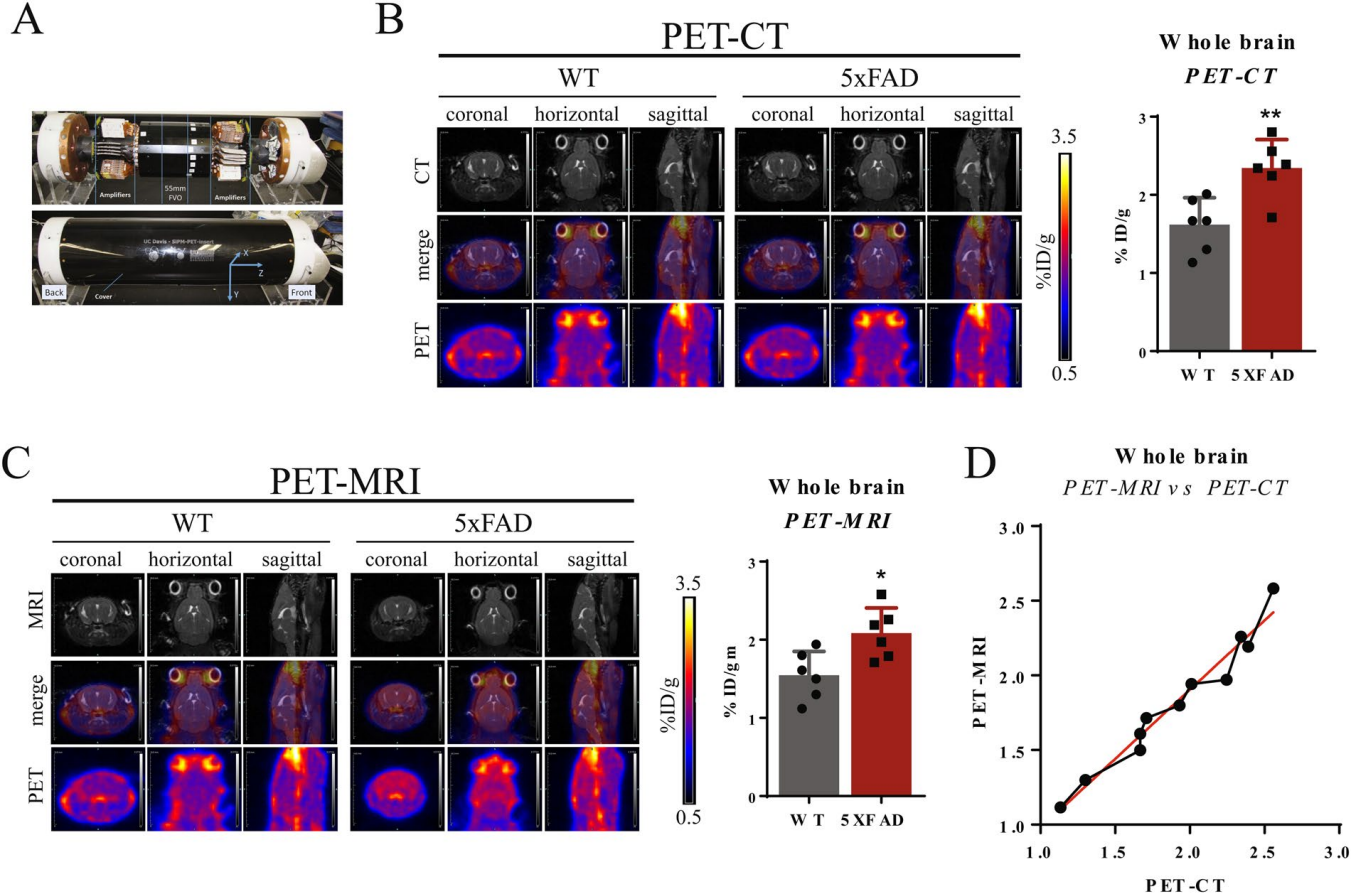
PET-CT and PET-MR scans are perfectly registered. **(A)** Photograph of PET insert utilized in this study. **(B)** Representative images of [^18^F]-Florbetapir uptake (coronal, horizontal, sagittal) for WT and 5xFAD mice by PET-CT (top: CT only, middle: PET + CT, bottom: PET only). White represents highest uptake, blue represents zero uptake. Uptake was quantified within the brain as percent injected dose gram (%ID/g). 5xFAD mice had significantly greater uptake than WT mice (unpaired Student’s *t*-test, p = 0.0144, n = 6). **(C)** Representative images of [^18^F]-Florbetapir uptake (coronal, horizontal, sagittal) for WT and 5xFAD mice by PET-MR (top: MRI only, middle: PET + MRI, bottom: PET only). White represents highest uptake, blue represents zero uptake. Uptake was quantified within the brain as percent injected dose / gram (%ID/g). 5xFAD mice had significantly greater uptake than WT mice (unpaired Student’s *t*-test, p = 0.0055, n = 6). MR scans provide much higher contrast and therefore anatomical detail compared to CT scans. (**D**) Correlation of %ID/gm for each individual mouse calculated by PET-MR (y-axis) and PET-CT (x-axis)(R^2^ = 0.96). All data are represented as mean ± SD. Images were analyzed using ASIpro software (Concorde Microsystems, Knoxville, TN, USA. https://www.sandersmedical.com/concordeMicro.htm) and the Inveon Research Workplace (IRW) software (Siemens Healthcare GmbH, Erlangen, Germany, https://www.siemens-healthineers.com/en-us/molecular-imaging/preclinical-imaging/preclinicalglobal-support).

We have developed a novel small-animal PET-MRI device (Fig. 1A) and imaged and quantified Aβ plaques in 5xFAD mice. We validated this system by comparing the tracer uptake in the whole brain (i.e., intracranial) measured by PET-MRI with whole-brain uptake measured by PET-CT immediately before the PET-MRI scan in the same mice. In addition, we quantified tracer uptake in multiple anatomical regions of the brain and demonstrated regional differences in tracer uptake, offering a reliable way to analyze brain region-specific amyloid deposition in AD mouse models.

## Results and discussion

### [^18^F]-Florbetapir uptake is significantly higher in the 5xFAD brain

We first assessed amyloid load in the whole brains (identified as intracranial activity) of 5xFAD mice compared to WT mice by measuring [^18^F]-Florbetapir uptake by PET-CT. Uptake was significantly greater in the 5xFAD whole brain (Fig. 1B) as has been previously reported (p = 0.0055). CT scanning (Fig. 1B, top panel) enables visualization of the skull to identify the brain but does not resolve specific brain regions. The PET scans for both PET-CT and PET-MRI are quantitative, meaning that they are parameterized in terms of percent of the injected dose per gram of tissue (%ID/g), with yellow to white pseudocolors corresponding to the highest %ID/g values and blue to black colors the lowest values. All PET images are thus quantitatively comparable. Of note, tracer uptake is very high in the eyes and area surrounding the skull of both WT and 5xFAD mice, as previously reported^26^. The registered and merged PET and CT images sets (Fig. 1B, middle panel) visually confirmed intracranial (i.e. brain) tracer uptake. WT mice have visually discernably lower tracer uptake within the brain than in the 5xFAD brain. Immediately after PET-CT scan, PET-MRI was performed in the same mice to measure uptake in the whole brain and sub-regions therein (Fig. 1C). The brain uptakes (in %ID/g) in the whole brain are shown (See Fig. 1D as well as the bar graphs in Figs. 1B,C). Here, MRI (Fig. 1C, top panel) was used to define total brain region. As with PET-CT, visually, WT mice have noticeably lower tracer accumulation within the brain, whereas 5xFAD mice have clear demonstrable uptake (Fig. 1C, middle panel). We quantified tracer uptake in the brain and determined a significant increase in 5xFAD as compared to WT mice (p = 0.0144). As noted, we quantified and compared the activity concentrations (in %ID/g) from the PET-CT and PET-MRI scans for each individual mouse and determined that the whole-brain activity concentrations determined by two scans had a highly correlated linear relationship, as expected (R^2^ = 0.96) (Fig. 1D). Although PET-CT could not be used to independently quantitate uptakes in sub-regions of the brain (because of the poor soft-tissue contrast of CT), PET-CT and PET-MRI clearly corroborated the whole-brain uptakes of [^18^F]-Florbetapir.

#### PET-MRI enables brain region specific analysis

**Figure 2:**
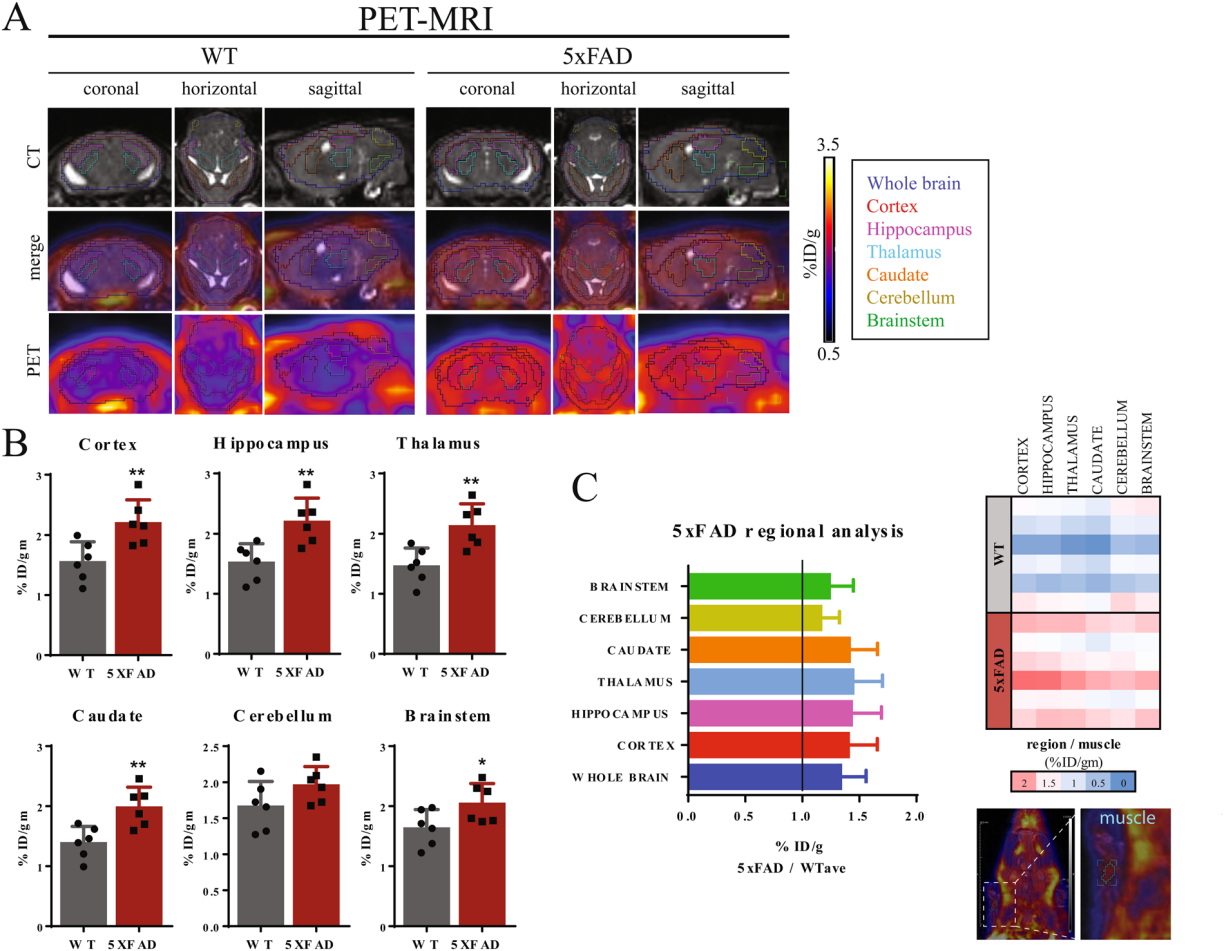
PET-MR enables brain region specific quantification of [18F]-Florbetapir uptake. **(A)** Representative images of [^18^F]-Florbetapir uptake (coronal, horizontal, sagittal) for WT and 5xFAD mice by PET-MRI (top: MRI only, middle: PET + MRI, bottom: PET only). White represents highest uptake, blue represents zero uptake. Specific regions outlined: whole brain (blue), cortex (red), hippocampus (pink), thalamus (aqua), caudate (orange), cerebellum (yellow), brainstem (green). (**B**) Uptake was quantified within each region of the brain as percent injected dose / gram (%ID/g). 5xFAD mice had significantly greater uptake than WT mice in the cortex, hippocampus, caudate, thalamus and brainstem, but not the cerebellum (unpaired Student’s *t*-test, p = 0.0094, p = 0.0065, p = 0.0061, p = 0.0054, p = 0.0451, respectively; n = 6). (**C**) (left) Average [^18^F]-Florbetapir uptake in each brain region of 5xFAD mice normalized to average WT uptake. (right) Heatmap of %ID/g in each brain region normalized to %ID/g in muscle in forelimb (representative ROI in blue) in the same mouse. Images were analyzed using ASIpro software the Inveon Research Workplace (IRW) software (see Figure legend).

We next identified 6 regions of interest (cortex, hippocampus, thalamus, caudate, cerebellum, brainstem) in the brain using the high structural resolution of the MRI scan and the Allen brain atlas (Fig. 2A, upper panel)^27^. Applying the MRI defined anatomical regions to PET image allows visualization and quantitation of uptake of [^18^F]-Florbetapir within these regions (Fig. 2A, bottom panel; Fig. 2B). As expected, the cortex and hippocampus had high uptakes in the 5xFAD mice compared to WT mice, whereas the differences in uptakes between the two groups in the brainstem and the cerebellum were much less (Fig. 2B). Interestingly, uptake in the thalamus and caudate was as high as that in the cortex and hippocampus. In fact, when normalized to average uptake in the WT mice, the thalamus had the highest ratio of uptake in 5xFAD compared to WT (1.46 ± 0.25) (Fig. 2C). In comparison, 5xFAD-to-WT uptake ratios (mean + SD) in the cortex, hippocampus and caudate mice was 1.42 ± 0.22, 1.44 ± 0.25 and 1.43 ± 0.23, respectively (p = 0.0094, p = 0.0065, p = 0.0061) (Fig. 2C). In contrast, uptakes in the brainstem and cerebellum of 5xFAD mice were only trending elevated above those in WT mice, with uptake ratios of 1.18 ± 0.15 and 1.25 ± 0.20, respectively (p = 0.0457, p = 0.1171) (Fig. 2C).

#### Post-mortem immunohistochemistry confirms high amyloid load in the cortex, hippocampus and thalamus

**Figure 3:**
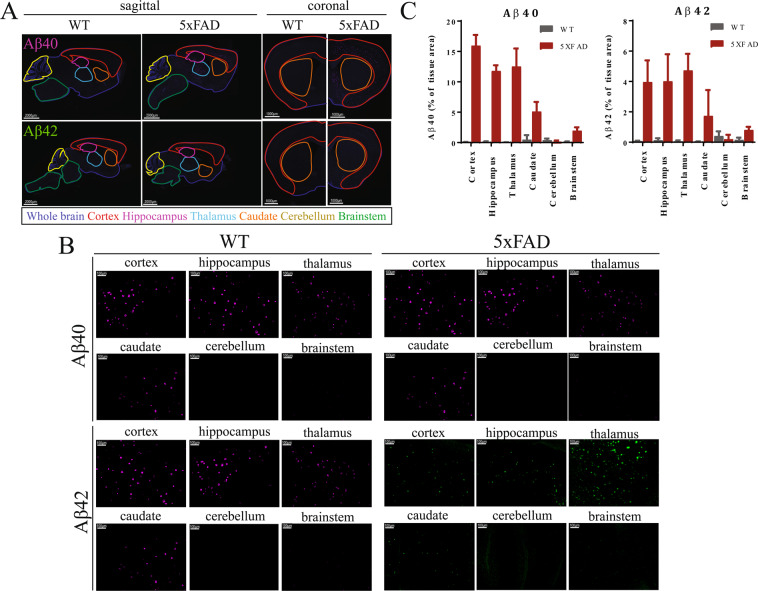
Immunohistochemical analysis confirms similar regional pattern of Aβ accumulation. **(A)** Fluorescence microscopy of Aβ40 expression in PFA-perfused mice (WT and 5xFAD). Representative images of sagittal sections (left) and coronal sections (right) show Aβ40 expression (pink – top panel) and Aβ42 expression (green – bottom panel). Scale bars represent 2000μm (sagittal) and 1000μm (coronal). Specific regions outlined: whole brain (blue), cortex (red), hippocampus (pink), thalamus (aqua), caudate (orange), cerebellum (yellow), brainstem (green). **(B)** Representative images of Aβ40 plaques (top panel) and Aβ42 plaques (bottom panel) within each region of WT (left panel) and 5xFAD (right panel) mice. Scale bars represent 100μm. **(C)** Fluorescence area for both Aβ40 and Aβ42 quantified as a percent of total area of each region (mean ± SD) (quantification from sagittal sections: cerebellum, brainstem, thalamus) (quantification from coronal sections: cortex, hippocampus, caudate).

After PET analysis, mice were perfused and their brains sectioned for Aβ staining. Half the mouse brains were sectioned sagittally and half coronally to allow for optimum visualization of each of the 6 brain regions. Sections were stained with specific antibodies against Aβ40 and Aβ42 to visualize both species. The same 6 anatomical regions of interest (cortex, hippocampus, thalamus, caudate, cerebellum, brainstem) were defined based on brain structures (Figs. 3A,B). Antibody signal in each region was quantified using FIJI (Fig. 3C). Amyloid staining was highest in the cortex, hippocampus and thalamus, and almost nil in the brainstem and cerebellum, corroborating our observations from the PET-MRI scans. The thalamus had the most intense Aβ42 staining and second most intense Aβ40 staining, supporting our PET-MRI-based conclusion that it is the region most vulnerable to Aβ accumulation. Notably, both Aβ40 staining and Aβ42 staining were comparatively weaker in the caudate than in the cortex, hippocampus and thalamus, which contrasts with tracer uptake quantified by PET-MRI. This discrepancy may be due to the use of Aβ species-specific antibodies versus assessing total Aβ load. If so, this would suggest differences in Aβ aggregation in the caudate as compared to other regions.

Using PET-MRI, we demonstrated region-specific differences in [^18^F]-Florbetapir uptake across the 5xFAD brain. Notably, we found high uptakes in the caudate and thalamus. Interestingly, our analysis agrees with that of PET-MRI scans of FAD patients. PIB retention is highest in the frontal and temporoparietal cortices of AD patient and there is negligible, non-specific retention in the cerebellum^12,13^. Amyloid deposition has been widely reported in the striatum and thalamus of AD patients^28–31^. Similarly, PIB retention has been demonstrated in the striatum of FAD patients who carry either the C410Y^32^ or A426P^33^ mutation in PS1 and late onset AD (LOAD) patients^34^. Retention of PIB was particularly notable in the cortical areas and in the anterior and ventral areas of the striatum in LOAD. While PS1C410Y patients did have PIB retention in the cortex and thalamus, it was strikingly high in the striatum, notably in the caudate putamen. A similar phenomenon of high striatal uptake of PIB binding and relatively less cortical retention as compared to LOAD patients was observed in PS1A426P carriers. This phenotype was confirmed with post-mortem plaque staining^34^. These differences between LOAD and FAD patients suggest that the striatum is most vulnerable to overproduction of amyloid in FAD patients, whereas the cortex is more effected by lack of clearance in LOAD patients.

To our knowledge, we are the first to utilize small-animal PET-MR for amyloid plaque imaging in an AD mouse model. Other methods, however, have been used to investigate region-specific tracer uptake. Recently, ^18^F-FC119S tracer was used to detect amyloid in 5.5-months-old 5xFAD mice. PET/CT images were spatially correlated with the Ma-Benveniste-Mirrione-T2 MR brain atlas to enable identification of ^18^F-FC119S uptake in the regions of hippocampus, cortex, thalamus and cerebellum. They found increased uptakes in the cortex, thalamus and hippocampus (33.3, 41.7 and 25.9% increases, respectively, but only hippocampal uptake increased significantly), but not in the cerebellum^35^. Furthermore, our findings are supported by direct MRI-based anatomic correlation. *In vivo*, non-contrast MRI of APP/PS1, PS1 and C57BL/6 mice determined that amyloid plaques located in the thalamus are more easily detectable than in any other region^36^.

The use of PET-MRI to measure pathology in AD mouse models has important implications. Previously use of PET-CT for quantification of tracer uptake across different brain regions required normalization to a brain atlas template. This approach can be limited in mouse models with neurodegeneration due to changes in brain anatomy and therefore, PET-MRI may be especially useful for tauopathy models. Furthermore, PET-MRI may be particularly useful in tracking if the localization of amyloid deposition changes overtime within the same mice and also whether amyloid localization is related to regional vulnerability to other pathologies such as gliosis and changes in cerebral blood flow^37,38^. Finally, PET-MRI could be combined with other technologies such as antibody-based radioligands for even greater specificity and resolution^39^. By using PET-MR, we can easily assess changes in AD pathology specifically in regions of interest such as the cortex, hippocampus and thalamus. This is vital for developing and testing novel therapeutics aimed at lowering Aβ load in mouse models and will also enable investigation of the progression of disease pathology using a clinically translatable imaging modality.

## Methods

### Mice

Six 14-month old C57BL/6J (WT) and six B6.CgTg(APPSwFlLon,PSEN1*M146L*L286V)6799Vas/Mmjax (5xFAD) mice were housed in compliance with the Institutional Animal Care and Use Committees (IACUC) of Memorial Sloan Kettering Cancer Center (MSKCC) guidelines. Tracer injections and scans were likewise performed in accordance with IACUC guidelines (the experimental protocols were approved by the IACUC of MSKCC, protocol 86-02-020). After scans, mice were anesthetized using a ketamine/xylazine cocktail and transcardially perfused with 4% paraformaldehyde (PFA) (the experimental protocol was approved by the IACUC of MSKCC, protocol 15-03-001).

### [^18^F]-Florbetapir (Amyvid) injections and anesthesia

[^18^F]-Florbetapir (Amyvid) was purchased ready-to-inject from PETNET Solutions, Inc. An activity of 600 to 800 μCi in a volume of ~200 μL was injected intravenously via a lateral tail vein. Following injection, animals were returned to their cages and remained awake until imaging at ~60 minutes post-injection. For imaging, animals were anesthetized by isoflurane inhalation (2.5% for induction and 1.5% for maintenance at a flow rate of 1.5 L/min with air as the carrier gas).

### PET/CT (work performed by MSKCC animal imaging core)

PET/CT images were acquired using the Inveon microPET/microCT (Siemens Healthcare GmbH, Erlangen, Germany) dedicated high-resolution small-animal PET scanner. This system provides isotropic PET spatial resolution of 1.7 mm FWHM and absolute activity quantitation (e.g. in %ID/gm).

### PET/MR (work performed by MSKCC animal imaging core)

The custom-built MR compatible PET scanner is designed to be compatible with Bruker 7 T Biospec scanner (Bruker BioSpin Corp., Billerica, MA) equipped with a 20-cm bore Bruker gradient with maximum 100 mT/m gradient strength. Novel Silicon photomultiplier (SiPM) arrays were used in this generation of the PET detectors for increased thermal stability and less MR sensitivity compared with the last-generation detectors^40^ based on avalanche photo diodes. The PET insert uses 4 rings of block detectors (10 crystals, 1.0 mm) rings covering an axial FOV of 55 mm and has a trans-axial FOV of 60 mm. Clinical PET processing electronics (Siemens Molecular Imaging, Knoxville, TN) are used to acquire data with high count rate performance. Custom data acquisition software was used for PET acquisition with an energy window of 350–650 keV. Simultaneous 3D MRI of the mouse head was acquired using a 300-MHz mouse volume coil (Doty Scientific, Columbia, SC, USA). Mouse head fast spin-echo RARE (Rapid Acquisition with Relaxation Enhancement) T2-weighted 3D MRI had a FOV of 6 × 3 × 2.5 cm and a spatial resolution of 234 × 234 × 260 μm, TR = 0.8 s and; TE = 47.3 ms with RARE factor of 16.

### PET/CT and PET/MRI data processing

For PET/MRI, PET images were processed off-line on a Linux workstation running custom-written 3D ordered sub-set expectation maximization (OSEM) reconstruction method^41^. Four iterations and 16 subsets were used in image reconstruction resulting in an image matrix of 64 × 64 × 64 and image resolution of 0.8 mm. Data were processed without attenuation, random coincidence, scatter, or dead-time corrections. PET and MRI image alignment were previously confirmed with a phantom. The resulting rigid-body transformation was then applied to the acquired animal MRI and PET datasets. Minor translational shifting was performed manually to achieve optimal alignment of the PET and MR scans.

PET-CT raw data were processed using the standard software provided by the manufacturers. PET data were acquired in list-mode, histogrammed by Fourier re-binning, and reconstructed using OSEM algorithm, with standard corrections for random coincidences, system response, and physical decay applied. The reconstructed PET images from both PET/CT and PET/MR scanners were quantitated using a measured system-specific ^18^F calibration factor to convert reconstructed count rates per voxel to activity concentrations (i.e., %ID/g). Manual tissue segmentation was done on co-registered 3D MR images. Brain subregions were delineated according to the Allen mouse brain atlas^27^. The regional ROIs were then used to calculate tissue radiotracer uptake from the reconstructed PET images (an additional ROI outside of the brain in the muscle of the forelimb was also used). The activity concentrations in the brain sub-regions were corrected for partial-volume averaging using measured correction factors (from phantom studies) and the MRI-derived dimensions of the sub-regions. No correction for attenuation or scatter was applied. Images were analyzed using ASIpro software (Concorde Microsystems, Knoxville, TN, USA. https://www.sandersmedical.com/concordeMicro.htm) and the Inveon Research Workplace (IRW) software (Siemens Healthcare GmbH, Erlangen, Germany, https://www.siemens-healthineers.com/en-us/molecular-imaging/preclinical-imaging/preclinicalglobal-support), which provides specialized 3D display and analysis capabilities. Both ASIPro and IRW were purchased from Concorde Microsystems and Siemens Healthcare, respectively, and no permission is required.

### Immunohistochemistry

#### Perfusion

Mice were anesthetized with a single dose of ketamine/xylazine (100 mg/kg/5.0 mg/kg) and transcardially perfused with 50 mL of PBS min followed by 50 mL of 4% paraformaldehyde (PFA). Brains were removed and post fixed in 4% PFA overnight at 4 °C then processed for paraffin embedding with tissue processor (Leica Biosystems, ASP6025) and 8-micron paraffin sections were obtained and mounted on slides for IHC.

#### Automated immunofluorescence staining (work performed at MSKCC molecular cytology core facility using discovery XT processor (ventana medical systems))

The tissue sections were deparaffinized with EZPrep buffer (Ventana Medical Systems) and antigen retrieval was performed with CC1 buffer (Ventana Medical Systems). Sections were blocked for 30 minutes with Background Buster solution (Innovex), followed by avidin-biotin blocking for 8 minutes (Ventana Medical Systems).

**G210:** Sections were incubated with anti-G210 (2ug/ml) for 5 hours, followed by 60-minute incubation with biotinylated horse anti-mouse IgG (Vector Laboratories, Inc. cat# MKB-2225B) at 1:200 dilution. The detection was performed with Streptavidin-HRP D (part of DABMap kit, Ventana Medical Systems), followed by incubation with Tyramide Alexa 488 (Invitrogen, cat# B40953) prepared according to manufacturer instruction with predetermined dilutions.

**10G3:** Sections were incubated with anti-10G3 (0.05ug/ml) for 5 hours, followed by 60-minute incubation with biotinylated horse anti-mouse IgG (Vector Laboratories, Inc. cat# MKB-2225B) at 1:200 dilution. The detection was performed with Streptavidin-HRP D (part of DABMap kit, Ventana Medical Systems), followed by incubation with Tyramide Alexa 488 (Invitrogen, cat# B40953) prepared according to manufacturer instruction with predetermined dilutions.

After staining, slides were counterstained with DAPI (Sigma Aldrich, cat# D9542, 5 ug/ml) for 10 min and cover-slipped with Mowiol. Slides were imaged with MIRAX SCAN (ZEISS). Caseviewer was used to annotate individual brain regions in the brain based on the Allen mouse brain atlas^27^. Image J software was used to quantify total fluorescence in these brain regions. The sagittally sectioned brains were used to quantify the cerebellum, brainstem and thalamus. The coronally sectioned brains were used to quantify the cortex, hippocampus and caudate.
