# Author Correction: Hybrid PET/MRI enables high-spatial resolution, quantitative imaging of amyloid plaques in an Alzheimer’s disease mouse model

**DOI:** 10.1038/s41598-020-70134-7

**Published:** 2020-08-11

**Authors:** Georgia R. Frost, Valerie Longo, Thomas Li, Lauren A. Jonas, Martin Judenhofer, Simon Cherry, Jason Koutcher, Carl Lekaye, Pat Zanzonico, Yue-Ming Li

**Affiliations:** 1 Chemical Biology Program, Memorial Sloan Kettering Cancer Center, New York, NY 10065 USA; 2 Program of Neuroscience, Weill Graduate School of Medical Sciences of Cornell University, New York, NY 10021 USA; 3 Program of Pharmacology, Weill Graduate School of Medical Sciences of Cornell University, New York, NY 10021 USA; 4 Small Animal Imaging Core Facility, Memorial Sloan Kettering Cancer Center, New York, NY 10065 USA; 5 Department of Biomedical Engineering, University of California, Davis, CA 95616 USA; 6 Departments of Medical Physics, Memorial Sloan Kettering Cancer Center, New York, NY 10065 USA; 7 Departments of Radiology, Memorial Sloan Kettering Cancer Center, New York, NY 10065 USA

Correction to: Scientific Reports, 10.1038/s41598-020-67284-z, published online 25 June 2020

This Article contains errors.

As a result of errors during preparation of the final versions of the figures, PET-CT part of Figure 1B is a duplication of PET-MRI part of Figure 1C. The correct Figure 1B is shown below as Figure 1.

**Figure 1:**
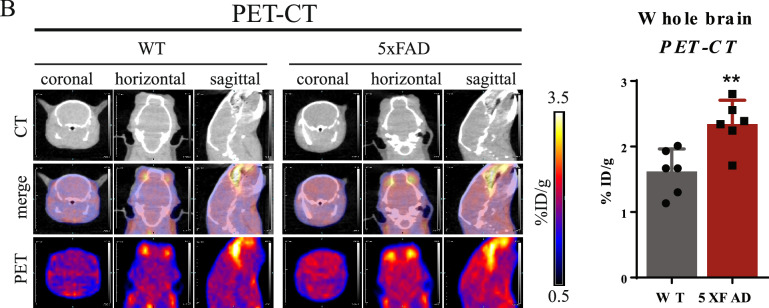
.

Additionally, some of the images in Figure 3B for the WT samples duplicate some of the images for the 5xFAD samples. The correct Figure 3B is shown below as Figure 2.

**Figure 2:**
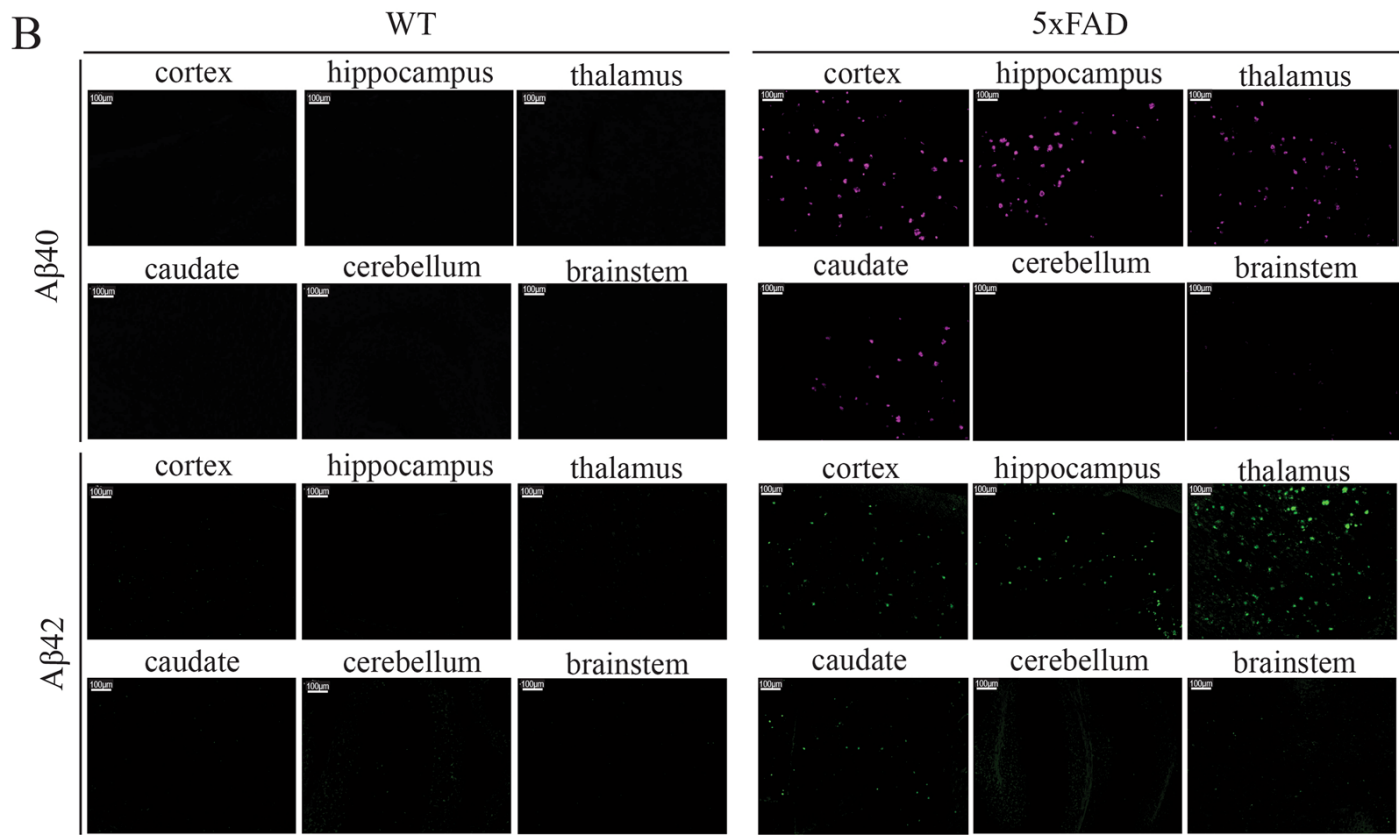
.

The correct data was included in Figures 1B and 3B during peer review. The bar chart in Figure 1B was correct at the time of publication.

These changes do not affect the conclusions of the Article.

